# Improvement of Image Binarization Methods Using Image Preprocessing with Local Entropy Filtering for Alphanumerical Character Recognition Purposes

**DOI:** 10.3390/e21060562

**Published:** 2019-06-04

**Authors:** Hubert Michalak, Krzysztof Okarma

**Affiliations:** Faculty of Electrical Engineering, West Pomeranian University of Technology, Szczecin, 70-313 Szczecin, Poland

**Keywords:** image binarization, optical character recognition, local entropy filter, thresholding, image preprocessing, image entropy

## Abstract

Automatic text recognition from the natural images acquired in uncontrolled lighting conditions is a challenging task due to the presence of shadows hindering the shape analysis and classification of individual characters. Since the optical character recognition methods require prior image binarization, the application of classical global thresholding methods in such case makes it impossible to preserve the visibility of all characters. Nevertheless, the use of adaptive binarization does not always lead to satisfactory results for heavily unevenly illuminated document images. In this paper, the image preprocessing methodology with the use of local image entropy filtering is proposed, allowing for the improvement of various commonly used image thresholding methods, which can be useful also for text recognition purposes. The proposed approach was verified using a dataset of 140 differently illuminated document images subjected to further text recognition. Experimental results, expressed as Levenshtein distances and F-Measure values for obtained text strings, are promising and confirm the usefulness of the proposed approach.

## 1. Introduction

Image binarization is one of the most relevant preprocessing steps leading to significant decrease in the amount of information subjected to further analysis and allowing for an increase of its speed. Such an operation is typically applied in many systems which utilize mainly shape recognition methods and do not require the colour or texture analysis. Some good examples might be some robotic applications, including line followers and visual navigation in corridors and labyrinths, advanced driver-assistance systems (ADAS) and autonomous vehicles with lane tracking, as well as widely used optical character recognition (OCR) methods. Binary image analysis may also be applied successfully in embedded systems with limited amount of memory and low computational power.

Nevertheless, the appropriate results of binary image analysis, in particular text recognition, depend on the correct prior binarization. In some applications, where the uniform illumination of the scene can be ensured, e.g., popular flatbed scanners or some non-destructive automated book scanners, even with additional infrared cameras allowing for software straightening the scanned book pages [1], the simplest global thresholding may be sufficient. However, in many other situations the illumination may be non-uniform, especially in natural images captured by cameras, and therefore more sophisticated adaptive methods should be applied.

One of the most challenging problems related to the influence of image thresholding on further analysis is document image binarization and therefore newly developed algorithms are typically validated by using intentionally prepared document images containing various distortions. For this reason well-known document image binarization competitions (DIBCO) datasets are typically used to verify the usefulness and validate the advantages of binarization methods. These databases are prepared for yearly document image binarization competitions organized during two leading conferences in this field—the International Conference on Document Analysis and the Recognition (ICDAR) [2] and International Conference on Frontiers in Handwriting Recognition (ICFHR) [3], where the H-DIBCO datasets are used, containing only handwritten document images without machine printed samples. All DIBCO datasets contain not only the distorted document images but also “ground truth” binary images and therefore the binarization results can be compared with them at the pixel level analysing the numbers of correctly and improperly classified pixels [4,5].

Despite the fact that image binarization is not a new topic, some enhancements of algorithms are still proposed, particularly for historical document image binarization, as well as unevenly illuminated natural images. A proposal of such an improvement based on the image entropy filter, possible to apply in many commonly known binarization methods, is presented in this paper.

The rest of the paper consists of the short overview of the most widely used image binarization methods, description of the proposed approach based on the use of local entropy filter, presentation and discussion of results and final conclusions.

## 2. Brief Overview of Image Binarization Algorithms

Probably the most popular image thresholding method was proposed in 1979 by Nobuyuki Otsu [6], who delivered the idea of minimizing the sum of intra-class variances of two groups of pixels classified as foreground and background, assuming the bi-modal histogram of the image pixels’ intensity. Hence, this approach leads to maximization of inter-class variance and therefore a good separation of two classes of pixels, represented finally as black and white, is achieved. Due to the operations on the histograms, this method is fast, although it works properly only for uniformly illuminated images with bi-modal histograms.

A similar approach, utilizing the entropy of the histogram instead of variances was proposed by Kapur et al. [7], whereas the idea of combining the global and local Otsu and Kapur methods was presented in the paper [8]. An extended adaptive version of Otsu method, known as AdOtsu, proposed by Moghaddam and Cheriet [9], assumed some additional operations such as multi-scale background estimation and calculation of average stroke widths and line heights. Since some images with unimodal histograms cannot be properly binarized using the above mentioned histogram-based methods another interesting idea was presented by Paul Rosin [10], who proposed to determine the threshold as the corner of the histogram curve.

Since the images containing some shadows being the result of non-uniform illumination should not be binarized using a single global threshold, some adaptive algorithms, which require the analysis of each pixels’ neighbourhood, were proposed as well. The most popular approach developed by Wayne Niblack [11] assumed the determination of the local threshold as the average local intensity lowered by the local standard deviation scaled by the constant parameter *k*. A further modification of this approach, utilizing the additional normalization of the local standard deviation by its division by its maximum value in the image, is known as Sauvola method [12]. Its multi-scale version was further developed by Lazzara and Géraud [13].

A simple choice of the local threshold as the average of the minimum and the maximum intensity within the local window (so called midgray value) was proposed by John Bernsen [14], whereas Bradley and Roth [15] developed the method using the integral image for the calculation of the local mean intensity of the neighbourhood. The implementation of this method, also in the modified versions utilising the local median and Gaussian weighted mean, is available as MATLAB *adaptthresh* function.

Some other adaptive binarization methods were proposed by Wolf and Jolion [16], who used a relatively simple contrast maximization approach as a modification of Niblack’s method, as well as Feng and Tan [17], where a similar idea based on the maximization of local contrast was used, however significantly slower due to the application of additional median filtering and bilinear interpolation. Another method proposed by Gatos et al. [18] utilizes a low-pass Wiener filtering and background estimation, followed by the use of Sauvola’s thresholding with additional interpolation and post-processing using so called shrink and swell filters to remove noise and fill some foreground gaps and holes.

More recent document image binarization methods include the idea of region-based thresholding using Otsu’s method with additional use of support vector machines (SVM) presented by Chou et al. [19] as well as faster region-based approaches [20,21]. Another method utilising the SVM-based approach with local features was presented recently by Xiong et al. [22].

The algorithm proposed by Howe [23] utilizes a Laplacian operator, Canny edge detection and graph cut method to find the threshold minimizing the energy. Erol et al. [24] proposed a more general approach related to the localization of text on a document captured by mobile phone camera using morphological operations for background estimation. Another background suppression method, although working properly mainly for evenly illuminated document images, was proposed by Lu et al. [25], whereas another attempt to the application of morphological operations was presented by Okamoto et al. [26].

Lelore and Bouchara [27] proposed the extended fast algorithm for document image restoration (FAIR) algorithm based on rough text localization and likelihood estimation followed by simple thresholding of the obtained super-resolution likelihood image. A multi-scale adaptive–interpolative method was proposed by Bag and Bhowmick [28], useful for faint characters. A method proposed by Su et al. [29] exploited adaptive image contrast map combined with results of Canny edge detection, whereas an attempt to use multiple thresholding methods was presented by Yoon et al. [30].

Some faster ideas of image thresholding based on the Monte Carlo method were proposed as well [31,32,33], where the simplified histogram of the image was approximated using the limited number of randomly chosen pixels. On the other hand, Khitas et al. [34] developed recently an algorithm based on median filtering used for estimation of the background information. An application of local features with Gaussian mixtures was examined in the paper [35], whereas Chen and Wang [36] used extended non-local means method followed by adaptive thresholding with additional postprocessing.

Bataineh et al. [37] developed an algorithm inspired by Niblack’s and Sauvola’s methods with additional application of dynamic windows. Further modifications of Niblack’s method were proposed by Khurshid et al. [38], Kulyukin et al. [39] and recently by Samorodova and Samorodov [40]. A direct binarization scheme of colour document images based on multi-scale mean-shift algorithm with the use of modified Niblack’s method was recently proposed by Mysoreet al. [41]. A review of many modifications of Niblack inspired algorithms can be found in Saxena’s paper [42], whereas many other approaches are discussed in some other survey papers [43,44,45]. Some earlier methods can also be found in *BinarizationShop* software developed by Deng et al. [46].

Some recent trends in image binarization are related to the use of variational models [47] and deep learning methods [48]. Recently, Vo et al. [49] proposed another supervised approach based on hierarchical deep neural networks. A comprehensive overview of many document image binarization algorithms can be found in the survey paper written by Sulaiman et al. [50].

An interesting method of binarization of non-uniformly illuminated images based on Curvelet transform followed by Otsu’s thresholding was proposed by Wen et al. [51]. However, the application of this algorithms requires the additional nonlinear enhancement functions and time-consuming multi-scale processing.

Some of the binarization methods utilize the calculation of histogram entropy as well as image entropy. The most widely known approach proposed by Kapur et al. [7] may be considered as the modification of the classical Otsu’s thresholding, which is based on earlier ideas presented by Thierry Pun [52,53]. Fan et al. [54] proposed a method maximizing the 2D temporal entropy, whereas Abutaleb [55] developed a method which uses pixel’s grey level as well the average of its neighbourhood for minimization of two-dimensional entropy. Brink and Pendock [56] used the cross-entropy instead of distance or similarity between the original image and the result of binarization to optimize the threshold. Some similar multilevel methods have been further developed as well for image segmentation [57], also with the use of genetic methods [58]. A ternary entropy-based method [59], based on the classification of pixels into text, near-text, and non-text regions was proposed as well, which utilized Shannon entropy, whereas Tsallis entropy was used by Tian and Hou [60]. Nevertheless, entropy-based methods are generally less popular than simple histogram-based thresholding or some adaptive binarization methods. Apart from the typical image binarization, one can find some other applications of entropy related to classification of signals or images obtained as the results of measurements or some other experiments, e.g., in a gearbox testing system presented by Jiang et al. [61], where Shannon entropy of the vibration signal is used to detect worn and cracked gears.

Development of any new image processing algorithms usually requires their reliable validation based on the comparison of the obtained results with the other methods. Stathis et al. [62] proposed a method of evaluation of binarization algorithms based on comparison of individual pixels, using the pixel error rate (PERR), peak signal to noise ratio (PSNR) and similar metrics, whereas some other approaches were presented in the survey paper by Sezgin and Sankur [63]. A much more popular approach is the use of typical classification metrics based on precision, recall, sensitivity, specificity or F-Measure [4,5], as well as the application of misclassification penalty metric (MPM) [64] or distance reciprocal distortion (DRD) [65]. Another binarization assessment method was presented by Lins et al. [66], which utilizes a dataset of synthetic images for comparison of various thresholding algorithms. Nevertheless, considering the final results of the document image recognition as the recognized text strings, a more useful approach would be the application of metrics calculated for characters instead of individual pixels. Apart from F-Measure, some metrics dedicated for text strings, such as Levenshtein distance, defined as the number of character operations necessary to convert one string into another, may be applied as well.

## 3. Proposed Method and Its Experimental Verification

### 3.1. Description of the Method

Analysing the unevenly illuminated document images, important information can be achieved with the use of the local image entropy, which may be calculated using the MATLAB entropyfilt function. Using its default parameters the local measure of randomness of the grey levels of the neighbourhood defined by the 9 × 9 pixels mask was achieved and stored as the result for the central pixel. Such an approach may be useful for image forgery detection, switching purposes in adaptive median filtering as well as for image preprocessing followed by comparison of properties of image regions. Hence, the local entropy filter was considered in the proposed method as one of the preprocessing steps for adaptive image binarization of unevenly illuminated document images subjected to further optical text recognition.

It is worth noting that most of the OCR engines used some “built-in” thresholding procedures and therefore their results are dependent also on the quality of the input data. For example, widely used freeware Tesseract OCR developed by Google utilized global Otsu’s thresholding, whereas the commercial ABBYY FineReader software employed the adaptive Bradley’s method. Therefore, the application of some other image binarization methods may improve or decrease the recognition accuracy, since the OCR “internal” thresholding does not change the input binary image. Hence, prior image thresholding may be considered as a replacement of the default methods used in the OCR engines.

The proposed method caused the equalization of illumination of an image, increasing also its contrast, making it easier to conduct the proper binarization and further recognition of alphanumerical characters. It is based on the analysis of the local entropy, assuming its noticeably higher values in the neighbourhood of the characters. Hence, only the relatively high entropy regions should be further analyzed as potentially containing some characters, whereas low entropy regions may be considered as the background. The proposed algorithm consists of the following steps:

- entropy filter—calculation of the local entropy using the predefined mask (in our experiments the most appropriate size is 19 × 19 pixels) leading to the local entropy map;
- negative—simple negation leads to more readable dark characters on a bright background; assuming the maximum entropy value equal to eight (considering eight bits necessary to store 256 grey levels), the additional normalization can be applied with the formula $Y=1-\frac{X}{8}$, where *X* is the local entropy map and the final range of the output image *Y* is 〈0;1〉;
- thresholding—one of the global binarization methods may be used for this purpose, in our experiments the classical Otsu’s thresholding was used, leading to the image *M* with segmented regions containing text and representing the background;
- masking—the obtained binary image *M* was used as the mask for the original input image, leading to the background image *B* with removed text regions;
- morphological dilation—the purpose of this operation was to fill the gaps containing the characters making it possible to obtain a full estimate of the background; a critical element of this step is an appropriate choice of the size of the structuring element (in our experiments the square 20 × 20 pixels one was sufficient and larger structuring elements caused an increase of the computation time);
- background subtraction—the expected result of the subtraction of the background estimate from the original input image should contain a bright text and the dark background with equalized illumination;
- negation with increase of contrast—a simple operation leading to the dark text and the bright background with improved readability;
- final binarization—the last step conducted after pre-processing, which can utilize any of commonly used binarization methods (in our experiments good results were obtained using adaptive Bradley’s and Niblack’s thresholding).

**Figure 1:**
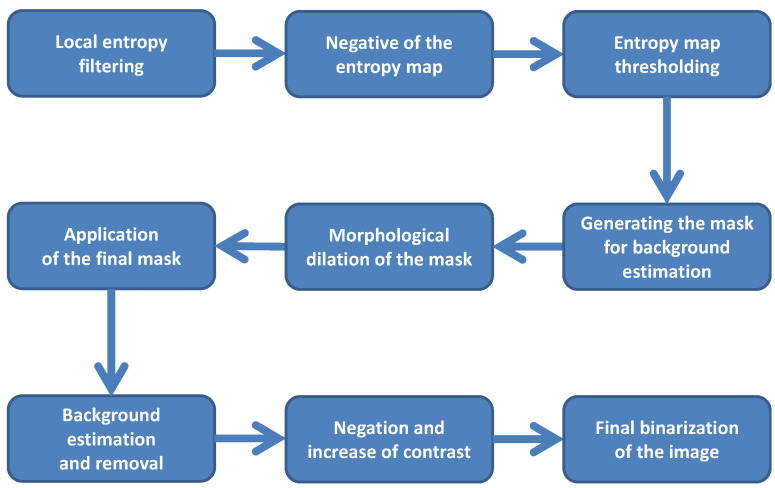
The simplified flowchart of the proposed method.

**Figure 2:**
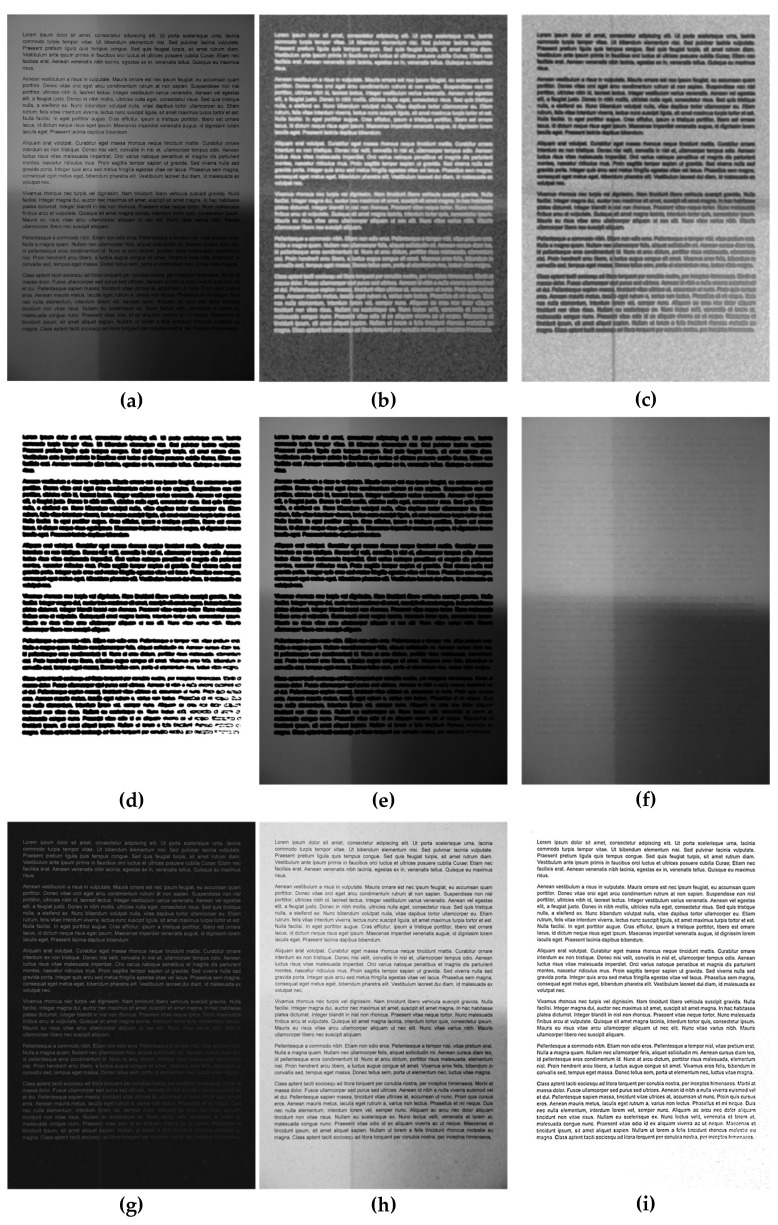
Results of the consecutive steps of the proposed algorithm obtained for an exemplary document image: (**a**) original input image, (**b**) local entropy map, (**c**) normalized negative entropy image, (**d**) binarized entropy image, (**e**) result of masking, (**f**) dilated masked image being the full background estimate, (**g**) result of background subtraction, (**h**) negative with eliminated background, and (**i**) final result of adaptive Niblack’s thresholding after preprocessing.

The simplified flowchart of the method is shown in Figure 1, whereas the illustration of results obtained after consecutive steps of the algorithm is presented in Figure 2.

### 3.2. Practical Verification

**Figure 3:**
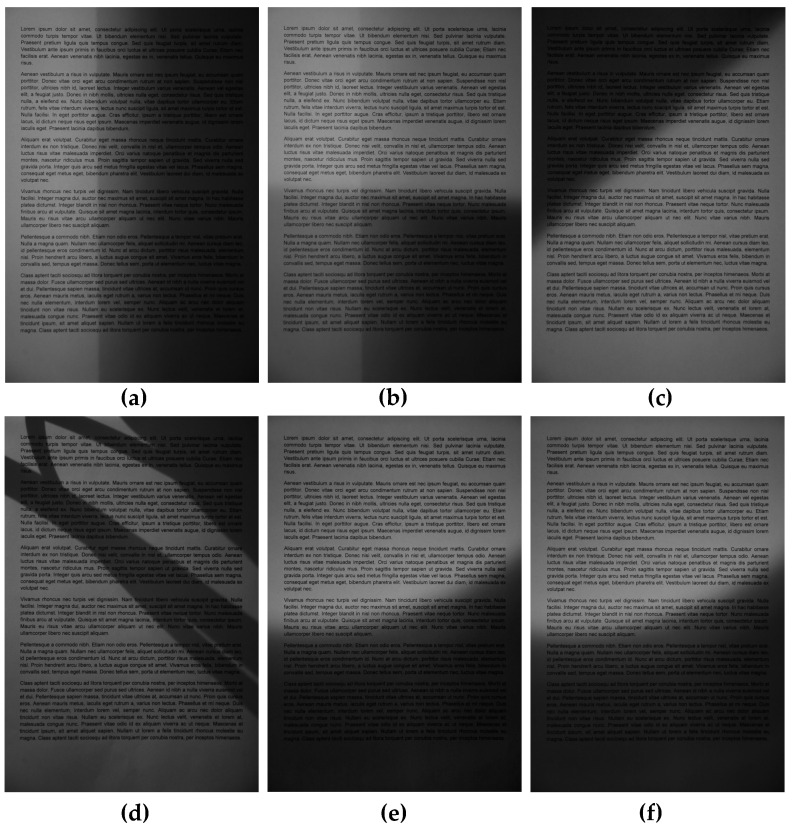
Exemplary unevenly illuminated images used in experiments: (**a**) side shading—series #2, (**b**) shading from the bottom—series #3, (**c**) diagonal shading—series #4, (**d**) irregular sharp shadow edges—series #5, (**e**) arc type shadows—series #6, (**f**) overexposure in the central part with underexposed boundaries—series #7.

The verification of the proposed method was conducted using the database of document images, prepared applying various illuminations (uniform lighting and six types of non-uniform or directional shadows). The well-known quasi-Latin text *Lorem ipsum*, used as the basis for the generated sample pages containing 536 words, was printed using five various font shapes (Arial, Times New Roman, Calibri, Verdana and Courier) and their style modifications (normal, bold, italics and bold+italics). Such printed 20 sheets of paper were photographed applying 7 types of illuminations mentioned above (six unevenly illuminated examples are shown in Figure 3). These 140 captured images were binarized in two scenarios: with and without the proposed preprocessing. In both cases several binarization algorithms were applied to verify the proposed approach in practice. All the obtained binary images were used as the input data for the Google Tesseract OCR engine. For each of the images, the number of correctly and incorrectly recognized characters were determined, allowing for the calculation of some typical classification metrics, such as F-Measure defined as:

(1) $$FM=2\cdot \frac{PR\cdot RC}{PR+RC},$$

where PR and RC stand for the precision (true positives to sum of all positives ratio) and recall (ratio of true positives to sum of true positives and false negatives). Hence, they can be expressed as:

(2) $$PR=\frac{TP}{TP+FP}\;\mathrm{and}\;RC=\frac{TP}{TP+FN},$$

where TP are true positives and FN false negatives, respectively. All positive and negative values are considered as the numbers of correctly and incorrectly recognized characters.

The additional metric, which may be applied for the evaluation of text similarity, is known as Levenshtein distance, representing the minimum number of text changes (insertions, deletions or substitutions of individual characters) necessary to change the analyzed text into another. This metric was also applied for evaluation purposes, assuming the knowledge of the original text string (Lorem ipsum-based in these experiments).

## 4. Results and Discussion

**Figure 4:**
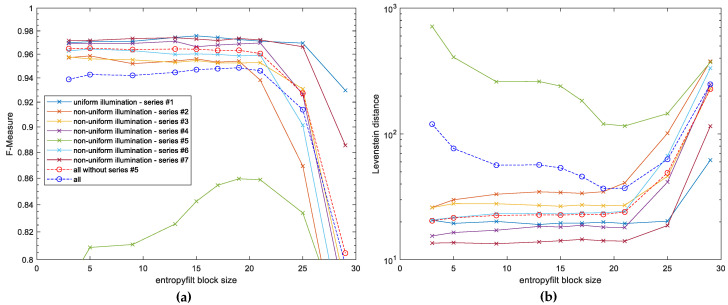
Experimental OCR results obtained for various size of blocks applied in the entropy filter: (**a**) F-Measure values, (**b**) Levenshtein distance.

The development of the final preprocessing algorithm allowing for the increase of the final OCR accuracy required an appropriate choice of some parameters mentioned earlier. The first of them is the size of the block used for the entropy filter which influences significantly the obtained results. Too small size of the filter would not be efficient due to its sensitivity to small details and noise whereas too big windows would be vulnerable to averaging effects. Since the default size of the filter in MATLAB entropyfilt function is 9 × 9 pixels, the first experiments were conducted using various windows to verify the influence of their size on the OCR results. The obtained results are presented in Figure 4, where the best values can be observed for 19 × 19 pixels filter. Therefore, the application of the default values would be inappropriate, particularly for the series #5 containing the non-uniformly illuminated images with sharp shadow edges as shown in Figure 3d.

**Figure 5:**
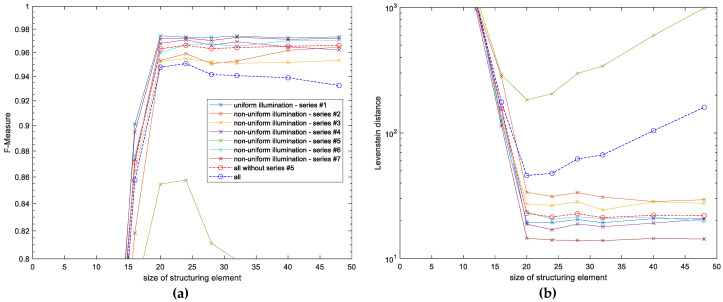
Experimental optical character recognition (OCR) results obtained for various size of structuring element applied for morphological dilation: (**a**) F-Measure values, (**b**) Levenshtein distance.

A similar difference may be observed during the choice of the most appropriate size of the structuring element applied during the morphological dilation, since the results obtained for the series #5 differ significantly from the others. Nevertheless, in all cases the choice of a similar size of the structuring element to the size of the block in the entropy filter leads to the best results as illustrated in Figure 5 (in our experiments 20 × 20 pixels structuring element was chosen).

**Figure 6:**
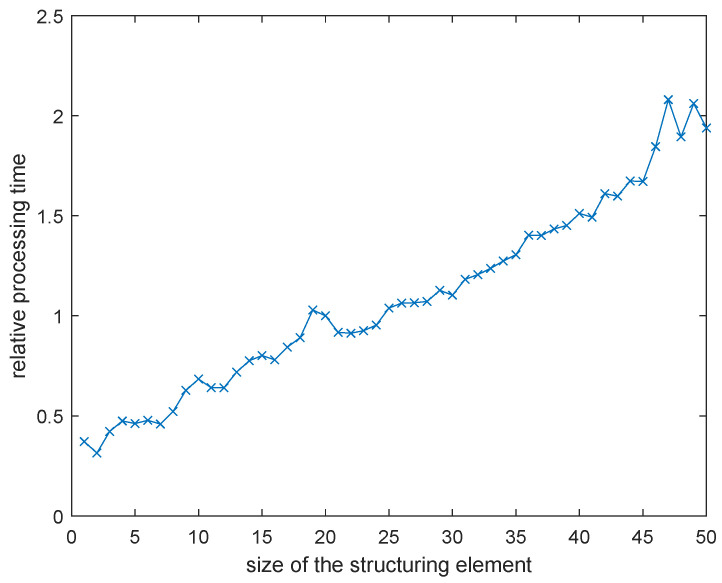
Normalized processing time for various size of structuring elements used in morphological dilation relatively to the time obtained applying the 20 × 20 pixels structuring element.

The additional reason of the choice of such structuring element was the processing time, which increased noticeably for bigger structuring elements as shown in Figure 6, where its values normalized according to the computation time obtained using the selected 20 × 20 pixels structuring element are presented. Unfortunately, relatively shorter processing did not guarantee good enough OCR accuracy, whereas increase of the structuring element’s size and computation time did not enhance the obtained results significantly. Since the experiments were conducted using a personal computer, some processes running in background (including the Tesseract OCR engine) might have influenced the obtained results. Nonetheless, the relation between the size of structuring element and the processing time can be considered as nearly linear. Hence, the most reasonable choice was the smallest possible structuring element not affecting the acceptable OCR accuracy level.

Having chosen the most appropriate parameters of the proposed preprocessing method, the obtained F-Measure values and Levenshtein distances for the whole dataset and each of the illumination types, as well as individual font faces and style modifications, were compared with some other methods applied without the proposed preprocessing. The comparison of the influence of the proposed preprocessing method on the F-Measure values is presented in Table 1, whereas respective Levenshtein distances are shown in Table 2. Analysing the results, a significant decrease of the Levenshtein distance, as well as the increase of the F-Measure values, may be observed for all methods, proving the usefulness of the proposed approach. The best results were achieved for Niblack, Sauvola and Wolf thresholding, as well as the simple Meanthresh method, which was significantly improved by the use of the entropy filtering-based preprocessing.

**Figure 7:**
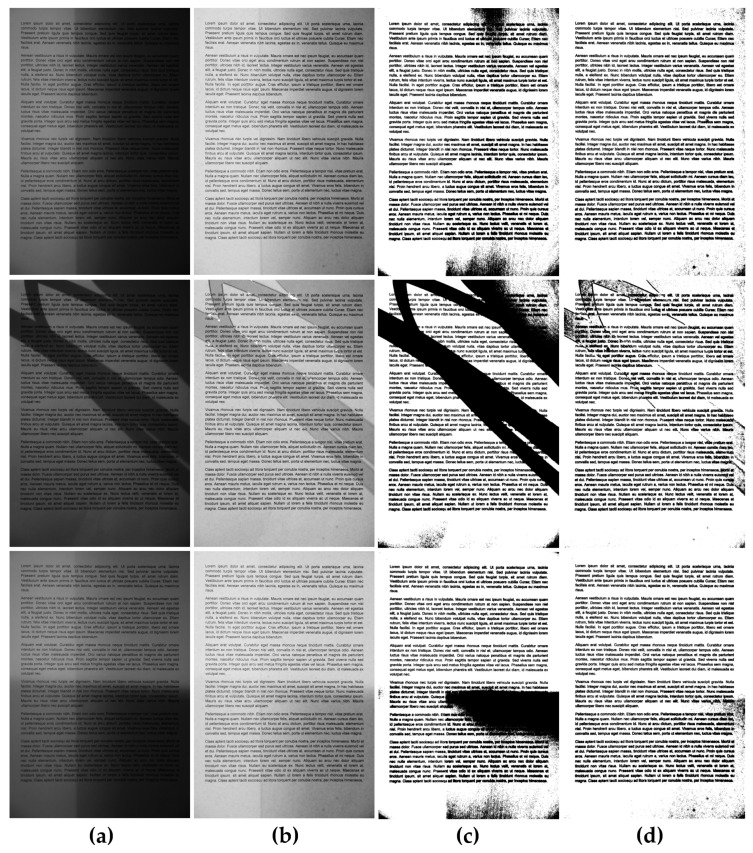
Comparison of binarization results obtained for exemplary unevenly illuminated images before the binarization: (**a**) without preprocessing, (**b**) with the proposed preprocessing, as well as using the Bradley method with a Gaussian kernel: (**c**) without preprocessing, (**d**) with the proposed preprocessing.

**Figure 8:**
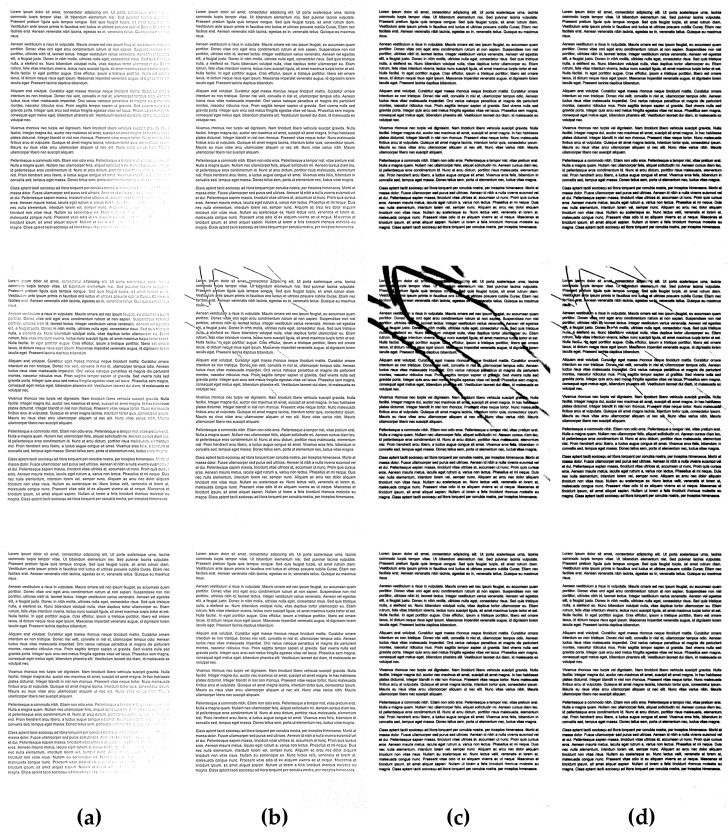
Comparison of binarization results obtained for exemplary unevenly illuminated images using the Niblack method: (**a**) without preprocessing, (**b**) with the proposed preprocessing, as well as Sauvola thresholding: (**c**) without preprocessing, (**d**) with the proposed preprocessing.

**Figure 9:**
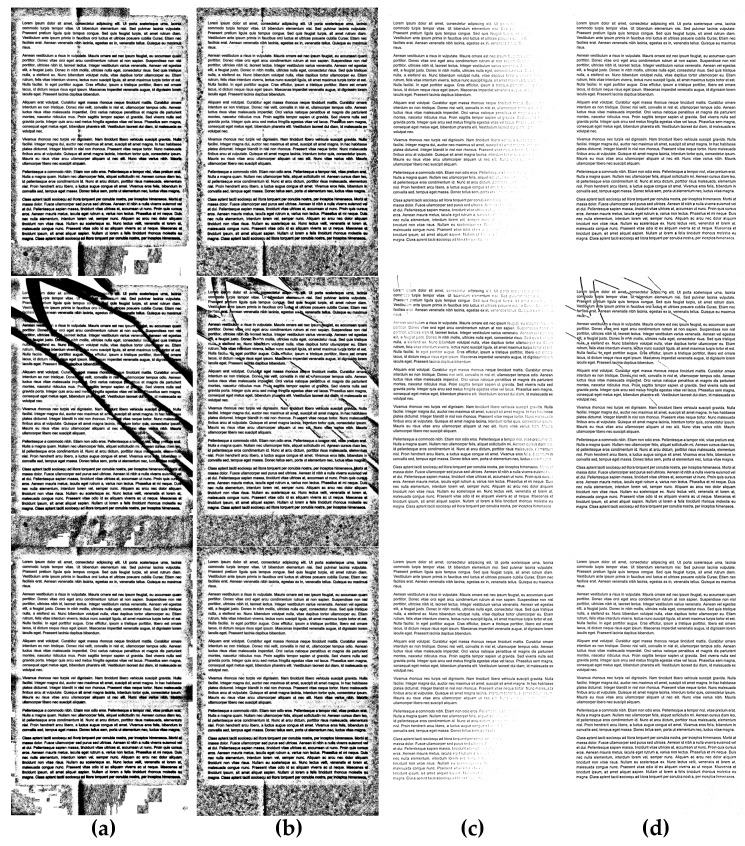
Comparison of binarization results obtained for exemplary unevenly illuminated images using the Bernsen method: (**a**) without preprocessing, (**b**) with the proposed preprocessing, as well as using the Meanthresh: (**c**) without preprocessing, (**d**) with the proposed preprocessing.

Some exemplary results obtained using the proposed preprocessing as well as its application for Bradley binarization with Gaussian kernel are illustrated in Figure 7. The additional illustration of its advantages for three exemplary images with the use of Niblack and Sauvola methods is shown in Figure 8, whereas another such comparison for Bernsen and Meanthresh methods is presented in Figure 9.

Since the properties of the proposed method may differ for various font shapes and styles, particularly for some of the thresholding algorithms, more detailed results are presented for them in Table 3 and Table 4, where F-Measure values can be compared for the same methods with and without the proposed entropy-based preprocessing method.

Comparing the influence of the proposed approach on the obtained OCR accuracy expressed as the F-Measure values calculated for individual text characters, relatively smaller enhancement may be observed for adaptive binarization methods, which achieve good results even without the proposed preprocessing method, such as Niblack or Sauvola. Nevertheless, in all cases the improvements may be noticed, also for the binarization method proposed by Wolf, which achieved much worse results for Courier fonts without the presented preprocessing method. A great improvement may also be observed for the simple mean thresholding as well as the direct usage of OCR engine’s built-in binarization, whereas the proposed method caused a small decrease of recognition accuracy after Bernsen thresholding for some font shapes (Courier and Times New Roman). It is worth to note that the proposed entropy-based preprocessing method always leads to better text recognition of bold fonts.

## 5. Conclusions

Binarization of unevenly illuminated and degraded document images is still an open and challenging field of research. Considering the necessity of fast image processing, many sophisticated methods, which cannot be effectively applied in many applications, may be replaced by simpler thresholding supported by less complicated preprocessing methods without the necessity of shape analysis or training procedures.

The approach proposed in the paper may be efficiently applied as the preprocessing step for many binarization methods in the presence of non-uniform illumination of document images, increasing significantly the accuracy of further text recognition, as shown in experimental results. Since its potential applicability is not limited to binarization of document images for OCR purposes, our further research may concentrate on the development of similar approaches for some other applications related to binarization of natural images and machine vision in robotics, particularly in unknown lighting conditions.

## Figures and Tables

**Table 1:** Comparison of F-Measure values obtained for various binarization methods with and without the proposed preprocessing.

Binarization Method	Series							
	#1	#2	#3	#4	#5	#6	#7	All
None	0.9638	0.6201	0.8139	0.6650	0.6693	0.7460	0.6260	0.7291
+ preprocessing	0.9728	0.6475	0.8729	0.7272	0.8167	0.8027	0.9584	0.8283
Otsu (global) [6]	0.9614	0.6281	0.7908	0.6662	0.6841	0.7598	0.6583	0.7355
+ preprocessing	0.9737	0.6400	0.8573	0.7312	0.8049	0.7947	0.9561	0.8226
Region-based [20]	0.9616	0.7579	0.8661	0.8407	0.7737	0.8318	0.9528	0.8550
+ preprocessing	0.9525	0.8377	0.8861	0.8254	0.7438	0.8468	0.9104	0.8575
Niblack [11]	0.9614	0.7920	0.8668	0.8444	0.8510	0.8567	0.9589	0.8759
+ preprocessing	0.9596	0.9439	0.9451	0.9516	0.8878	0.9436	0.9674	0.9427
Sauvola [12]	0.9709	0.9581	0.9646	0.9722	0.7660	0.9655	0.9721	0.9385
+ preprocessing	0.9674	0.9635	0.9665	0.9668	0.8401	0.9671	0.9694	0.9487
Wolf [16]	0.9661	0.9482	0.9513	0.9514	0.7614	0.9594	0.9703	0.9297
+ preprocessing	0.9691	0.9661	0.9643	0.9662	0.8561	0.9621	0.9657	0.9499
Bradley (mean) [15]	0.9665	0.9191	0.9093	0.8484	0.7369	0.8976	0.9699	0.8925
+ preprocessing	0.9666	0.8896	0.9169	0.9262	0.8040	0.9103	0.9642	0.9111
Bradley (Gaussian) [15]	0.9663	0.8521	0.8295	0.7528	0.7267	0.7907	0.9489	0.8381
+ preprocessing	0.9678	0.8863	0.8991	0.8741	0.7521	0.8786	0.9124	0.8815
Feng [17]	0.9110	0.3782	0.7924	0.6312	0.7292	0.7938	0.8461	0.7285
+ preprocessing	0.9261	0.4418	0.7990	0.6489	0.7103	0.8076	0.8688	0.7432
Bernsen [14]	0.6948	0.6414	0.6844	0.6467	0.6286	0.7122	0.7245	0.6764
+ preprocessing	0.6971	0.6688	0.6938	0.6752	0.6312	0.7047	0.7141	0.6836
Meanthresh	0.9597	0.7348	0.8314	0.7921	0.8317	0.7947	0.9308	0.8393
+ preprocessing	0.9651	0.9570	0.9596	0.9602	0.8970	0.9606	0.9684	0.9525

**Table 2:** Comparison of Levenshein distances obtained for various binarization methods with and without the proposed preprocessing.

Binarization Method	Series							
	#1	#2	#3	#4	#5	#6	#7	All
None	56.40	1897.20	1031.80	1362.40	1548.30	1387.90	1815.50	1299.93
+ preprocessing	10.90	1665.10	718.40	1045.20	512.55	1063.85	68.15	726.31
Otsu (global) [6]	62.75	1878.20	1039.80	1393.40	1514.55	1358.55	1715.80	1280.44
+ preprocessing	12.60	1671.85	720.05	1047.05	514.20	1066.75	76.10	729.80
Region-based [20]	27.30	537.40	388.35	217.50	294.35	423.60	44.75	276.18
+ preprocessing	27.40	133.55	78.90	141.60	378.55	166.30	48.15	139.21
Niblack [11]	30.50	560.55	359.95	388.00	222.10	398.05	31.15	284.33
+ preprocessing	26.00	42.90	35.40	25.55	79.45	32.20	16.55	36.86
Sauvola [12]	20.30	22.85	17.35	14.80	651.60	17.75	12.40	108.15
+ preprocessing	22.40	30.25	23.05	17.35	197.55	19.75	15.95	46.61
Wolf [16]	21.35	54.90	69.90	74.05	923.65	58.55	17.60	174.29
+ preprocessing	21.45	27.75	19.75	23.50	202.75	17.80	16.65	47.10
Bradley (mean) [15]	26.45	63.15	157.15	389.45	1231.95	188.25	17.35	296.25
+ preprocessing	26.30	75.60	52.05	44.10	312.15	54.05	19.05	83.33
Bradley (Gaussian) [15]	27.10	355.80	731.00	950.75	1282.40	1136.70	32.25	645.14
+ preprocessing	25.75	91.05	193.00	219.95	700.15	149.50	19.95	199.91
Feng [17]	66.20	2518.00	1069.50	1507.50	1030.50	1037.10	174.20	1057.57
+ preprocessing	59.15	2385.25	1015.75	1435.10	887.75	945.70	142.30	981.57
Bernsen [14]	467.75	1471.25	1071.00	1273.65	1634.15	1167.40	623.10	1101.19
+ preprocessing	490.40	1178.10	1046.75	1011.85	1402.25	1093.35	687.35	987.15
Meanthresh	20.85	776.30	529.10	519.85	250.70	763.35	72.40	418.94
+ preprocessing	21.95	26.10	21.55	17.25	81.65	20.00	14.20	28.96

**Table 3:** Comparison of F-Measure values obtained for various binarization methods with and without the proposed preprocessing for various font faces.

Binarization Method	Font Face				
	Arial	Times New Roman	Calibri	Courier	Verdana
None	0.7556	0.7432	0.7374	0.6483	0.7612
+ preprocessing	0.8541	0.8277	0.7886	0.8173	0.8539
Otsu (global) [6]	0.7489	0.7528	0.7525	0.6598	0.7637
+ preprocessing	0.8506	0.8214	0.7802	0.8058	0.8548
Region-based [20]	0.8726	0.8799	0.8738	0.7970	0.8514
+ preprocessing	0.8513	0.8689	0.8590	0.8425	0.8659
Niblack [11]	0.8776	0.9012	0.8729	0.8499	0.8777
+ preprocessing	0.9463	0.9550	0.9475	0.9195	0.9452
Sauvola [12]	0.9395	0.9476	0.9412	0.9239	0.9402
+ preprocessing	0.9555	0.9540	0.9450	0.9395	0.9495
Wolf [16]	0.9399	0.9507	0.9355	0.8826	0.9400
+ preprocessing	0.9567	0.9558	0.9551	0.9310	0.9511
Bradley (mean) [15]	0.9036	0.9004	0.8946	0.8676	0.8963
+ preprocessing	0.9158	0.9186	0.9158	0.8906	0.9147
Bradley (Gaussian) [15]	0.8475	0.8448	0.8434	0.8087	0.8463
+ preprocessing	0.9004	0.8992	0.8873	0.8609	0.8599
Feng [17]	0.7137	0.7430	0.7113	0.7528	0.7210
+ preprocessing	0.7368	0.7462	0.7304	0.7540	0.7487
Bernsen [14]	0.6735	0.6970	0.6938	0.6213	0.6971
+ preprocessing	0.7062	0.6917	0.6956	0.6041	0.7202
Meanthresh	0.8251	0.8698	0.8483	0.8197	0.8337
+ preprocessing	0.9511	0.9623	0.9516	0.9429	0.9548

**Table 4:** Comparison of F-Measure values obtained for various binarization methods with and without the proposed preprocessing for various font styles.

Binarization Method	Font Style			
	Normal	Bold	Italic	Bold + Italic
None	0.6945	0.7497	0.7221	0.7291
+ preprocessing	0.8049	0.8455	0.8076	0.8283
Otsu (global) [6]	0.7095	0.7544	0.7272	0.7355
+ preprocessing	0.7980	0.8426	0.8038	0.8226
Region-based [20]	0.8631	0.8444	0.8700	0.8550
+ preprocessing	0.8621	0.8590	0.8593	0.8575
Niblack [11]	0.8781	0.8898	0.8669	0.8759
+ preprocessing	0.9396	0.9444	0.9424	0.9427
Sauvola [12]	0.9366	0.9377	0.9340	0.9385
+ preprocessing	0.9464	0.9545	0.9463	0.9487
Wolf [16]	0.9165	0.9430	0.9223	0.9297
+ preprocessing	0.9428	0.9567	0.9467	0.9499
Bradley (mean) [15]	0.8888	0.8942	0.8916	0.8925
+ preprocessing	0.9031	0.9230	0.9099	0.9111
Bradley (Gaussian) [15]	0.8342	0.8418	0.8370	0.8381
+ preprocessing	0.8801	0.8738	0.8754	0.8815
Feng [17]	0.7333	0.7342	0.7391	0.7285
+ preprocessing	0.7368	0.7458	0.7518	0.7432
Bernsen [14]	0.6722	0.6786	0.6718	0.6764
+ preprocessing	0.6656	0.7060	0.6573	0.6836
Meanthresh	0.8379	0.8454	0.8381	0.8393
+ preprocessing	0.9547	0.9519	0.9541	0.9525

## Abbreviations

The following abbreviations are used in this manuscript:

ADAS	advanced driver-assistance system
DIBCO	document image binarization competition
DRD	distance reciprocal distortion
FAIR	fast algorithm for document image restoration
ICDAR	International Conference on Document Analysis and Recognition
ICFHR	International Conference on Frontiers in Handwriting Recognition
MPM	misclassification penalty metric
OCR	optical character recognition
PERR	pixel error rate
PSNR	peak signal to noise ratio
SVM	support vector machines
